# Disease-related Huntingtin seeding activities in cerebrospinal fluids of Huntington’s disease patients

**DOI:** 10.1038/s41598-020-77164-1

**Published:** 2020-11-20

**Authors:** C. Y. Daniel Lee, Nan Wang, Koning Shen, Matthew Stricos, Peter Langfelder, Kristina H. Cheon, Etty P. Cortés, Harry V. Vinters, Jean Paul Vonsattel, Nancy S. Wexler, Robert Damoiseaux, Judith Frydman, X. William Yang

**Affiliations:** 1 Center for Neurobehavioral Genetics, The Jane and Terry Semel Institute for Neuroscience & Human Behavior, University of California, Los Angeles, Los Angeles, USA; 2 Department of Psychiatry and Biobehavioral Sciences, David Geffen School of Medicine at UCLA, Los Angeles, CA USA; 3 Department of Biology and BioX Program, Stanford University, Stanford, CA USA; 4 Division of Aging and Dementia, Department of Neurology, Columbia University Medical Center, New York, NY USA; 5 Department of Pathology and Laboratory Medicine, Neurology, David Geffen School of Medicine at UCLA, Los Angeles, CA USA; 6 Departments of Neurology and Psychiatry, College of Physicians and Surgeons, Columbia University, New York, NY USA; 7 Hereditary Disease Foundation, New York, NY USA; 8 California NanoSystems Institute, University of California, Los Angeles, CA USA; 9 Department of Molecular and Medical Pharmacology, University of California, Los Angeles, CA USA; 10 Present Address: Department of Molecular and Cell Biology, UC Berkeley, Berkeley, CA USA

**Keywords:** Huntington's disease, Diagnostic markers, Diseases, Neurology

## Abstract

In Huntington’s disease (HD), the mutant Huntingtin (mHTT) is postulated to mediate template-based aggregation that can propagate across cells. It has been difficult to quantitatively detect such pathological seeding activities in patient biosamples, e.g. cerebrospinal fluids (CSF), and study their correlation with the disease manifestation. Here we developed a cell line expressing a domain-engineered mHTT-exon 1 reporter, which showed remarkably high sensitivity and specificity in detecting mHTT seeding species in HD patient biosamples. We showed that the seeding-competent mHTT species in HD CSF are significantly elevated upon disease onset and with the progression of neuropathological grades. Mechanistically, we showed that mHTT seeding activities in patient CSF could be ameliorated by the overexpression of chaperone DNAJB6 and by antibodies against the polyproline domain of mHTT. Together, our study developed a selective and scalable cell-based tool to investigate mHTT seeding activities in HD CSF, and demonstrated that the CSF mHTT seeding species are significantly associated with certain disease states. This seeding activity can be ameliorated by targeting specific domain or proteostatic pathway of mHTT, providing novel insights into such pathological activities.

## Introduction

Huntington’s disease (HD) is a dominantly inherited neurodegenerative disorder caused by CAG repeat expansion encoding a polyglutamine (polyQ) stretch near the N-terminus of mutant Huntingtin (mHTT). The mutant polyQ repeat increases the propensity of mHTT to misfold and form aggregates in the striatum and cortex of HD patients^1^. The precise pathogenic roles of the mHTT aggregates remain elusive. Certain studies suggest that they may be protective^2^ or neutral^3,4^, while others show that they are pathogenic^5–7^ and possibly contribute to the selective vulnerability of striatal medium spiny neurons (MSNs) in HD^8^.

Several antibody-based platforms have been developed to quantitatively detect HTT species as a biomarker in biosamples^9,10^. The Singulex single-molecule counting immunoassay (SMC) can detect a significant increase of HTT in cerebrospinal fluids (CSF) and plasma from premanifest to manifest HD stages^9,10^. Importantly, this assay has been successfully employed in the antisense oligonucleotide (ASO) trial in HD patients to show the reduction of mHTT in the CSF of HD patients^11^. The ELISA-based meso-scale discovery (MSD) electrochemiluminescence assay is a platform that sensitively quantifies and differentiates soluble and aggregated mHTT in brain tissues and blood cells^12–14^. The microbead-based immunoprecipitation and flow cytometry assay (IP-FCM) has been shown to detect mHTT in the brain of HD mouse models and CSF of patients^15^. The study further showed that ASO against HTT could reduce CSF mHTT in BACHD, a mouse model of HD^15,16^. These sensitive antibody-based methods demonstrated the presence of mHTT in the more accessible patient biofluids. However, since HTT is a large protein (350 kDa) with extensive post-translational modifications and aggregates to form higher order structures^17,18^, it is likely that these antibody-based assays may capture a subset (e.g. specific conformations) of mHTT species.

Emerging studies show that pathogenic proteins in a variety of neurodegenerative disorders may exhibit “prion-like” properties, leading to the spreading and template-based amplification of misfolded disease proteins (e.g. aggregated α-synuclein in Parkinson’s disease and hyperphosphorylated Tau in Tauopathy and Alzheimer’s disease)^19,20^. This is postulated to be a key mechanism in disease progression^19,20^. Previous studies reported that the aggregation of recombinant mHTT fragments in vitro could be greatly accelerated by the addition of preformed mHTT aggregates^21^, a phenomenon often referred to as mHTT “seeding activities”. Moreover, extracellular application of preformed mHTT aggregates or crude extracts of aggregates from postmortem HD brains or mouse models of HD could also seed intracellular aggregation of otherwise soluble forms of HTT^22–25^. One later study demonstrated that extracts from modified PC12 cells expressing inducible GFP-tagged mHTT-exon 1 fragment with 103Q showed increased aggregation with the addition of CSF from HD patients^26^. It sheds the light of using seed-induced aggregation for detecting mHTT seeding species in the biosamples. However, this assay is limited by its sensitivity and scalability since the measurements of induced aggregation rely on cell counting and filter retardation assays, and the cell line used in this study spontaneously forms reporter protein aggregates without the addition of extracellular mHTT seeds^26^.

In this study, we developed cell lines stably expressing wildtype and engineered mHTT-exon 1 reporters. We found that deleting the N17 domain of mHTT-exon 1 greatly augments the sensitivity of the reporter cells for detection mHTT seeding species. Importantly, with CSF samples from postmortem brains and living HD patients, we showed that the seedable mHTT species in the patient CSF increased with the progression of clinical disease presentation and neuropathology. Moreover, we showed the CSF mHTT seeding activities can be abolished by overexpressing the chaperone DNAJB6 and by antibodies against the polyproline domain of HTT. These results suggest the presence and level of seeding-competent mHTT species in the patient CSF may correlate with neuropathological progression, and substantiate the use of this highly sensitive and scalable cell-based assay for studying mHTT seeding activities in HD clinical progression and response to therapy.

## Results

### Deleting the N17 domain of mHTT significantly enhanced the sensitivity of the reporter cells to detect mHTT seeding species

**Figure 1:**
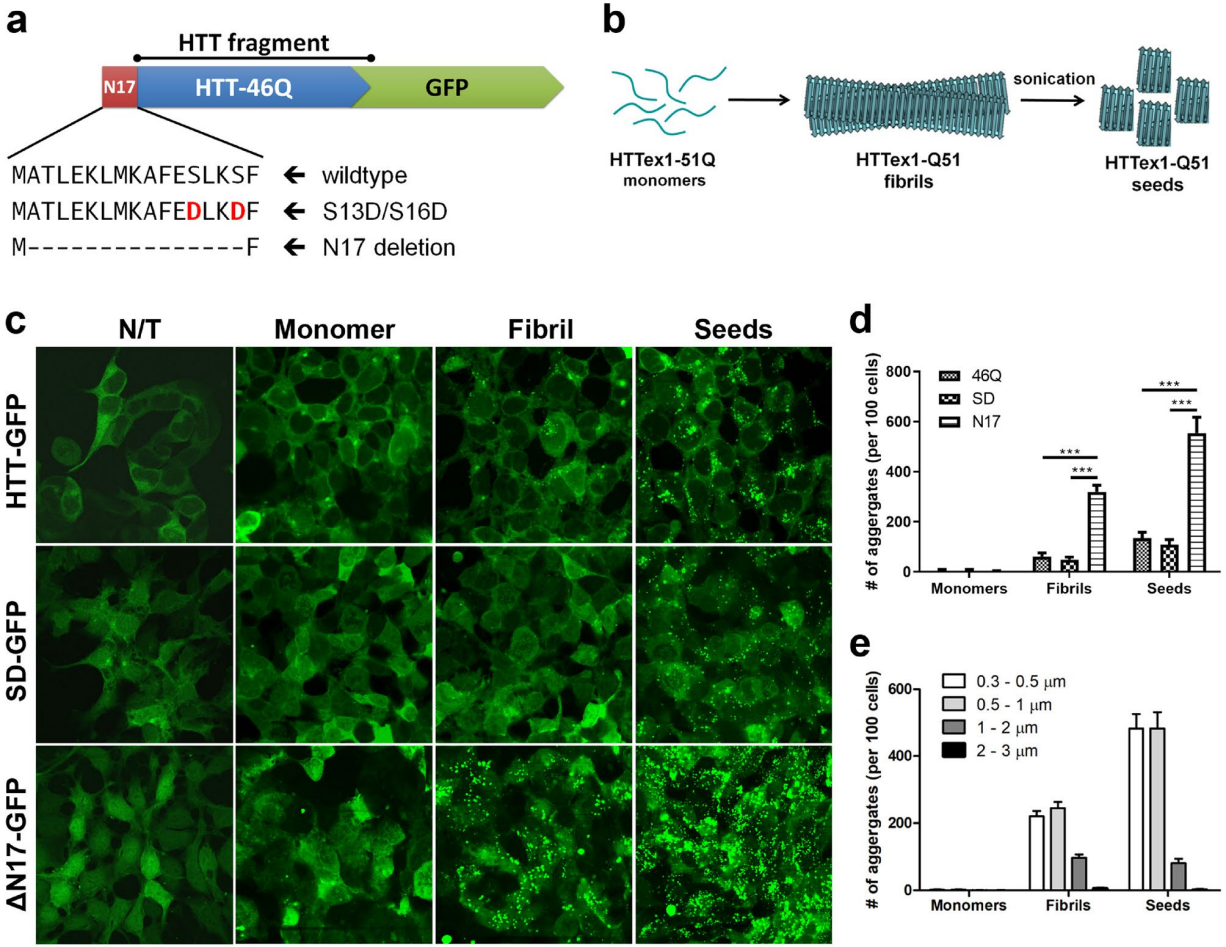
N17 mutations in recipient cell lines altered sensitivity of seeding-induced mHTT aggregation. **(a)** A diagram illustrates the constructs of GFP-tagged mHTT-exon 1 (46Q) fragments, which are used to generate stable cell lines. (**b**) A diagram illustrates the generation of mHTT aggregates (fibrils) and seeds from monomeric recombinant mHTT-51Q proteins. (**c**) Cell lines generated by stably transfected HEK-293 cells with GFP tagged mHTT-exon 1 (46Q) variants were used as recipient cells of the seeding assay. Constructs of the transgenes are labeled on the left of the panel. Recombinant mHTT-exon 1 (51Q) in the forms of monomers, fibrils and seeds were added directly to the cultured cells for 3 days. Representative confocal images of the cells are shown. Bright green punctae are intracellular mHTT-GFP aggregates induced by seeding. N/T = non-treated. (**d**,**e**) The number of HTT-GFP aggregates was quantified using ImageJ and presented as mean ± s.e.m., *n* = 5–10, **p < 0.01.

Prior studies showed that reporter cells expressing fluorescent proteins tagged mHTT fragment with either an expanded pathogenic repeat or a short nonpathogenic repeat could be used as intracellular reporters for exogenous “mHTT seeds” induced aggregation^22,26^. However, these cell lines fall short of sensitivity, specificity and scalability in detecting the presence of mHTT seeds in HD patient biosamples, such as CSF. To develop a reporter cell line that fulfill these requirements, we reasoned that the cells should stably express a mHTT N-terminal fragment with a moderately expanded polyQ repeat, which thus could be nucleated to form aggregates while not spontaneously aggregates at the baseline. We first generated a stable HEK293 cell line expressing mHTT-exon 1 fragment with 46Q and in-frame C-terminal green fluorescent protein fusion (mHTT-46Q-GFP; Fig. 1a). We chose this modestly expanded polyQ repeat, which is within the range of the common adult-onset HD patients^27^. It is metastable and can form oligomers or small aggregates in neurons. Unlike mHTT-exon 1 expression constructs with over 100Q^2,28^, mHTT-46Q peptides rarely form aggregates when expressed at a moderate level in cultured cells^29^. Consistent with this observation, we found that HEK293 cells stably expressing mHTT-46Q-GFP did not show visible aggregates at the baseline (Fig. 1c). We treated this cell line with purified recombinant mHTT-exon 1 with 51Q (mHTT-51Q) in the forms of monomers, fibrils and sonicated fibrils (also referred as mHTT seeds hereafter; Φ = 30.7–49.6 nm; Fig. 1b)^30^. We found increasing levels of GFP^+^ aggregates in reporter cells treated with extracellular mHTT fibrils and seeds, while monomeric mHTT-51Q did not induce mHTT-46Q-GFP aggregation (Fig. 1c).

Since the first 17 amino acid (N17) of mHTT is critical in regulating the subcellular localization, oligomerization and aggregation, and toxicities of mHTT in vitro and in vivo^29–31^, we tested whether mutations in the N17 domain influenced the sensitivity of the reporter cells in detecting extracellular mHTT fibrils and seeds. To this end, we developed cell lines stably expressing mHTT-exon 1-46Q-GFP reporters with two distinct N17 mutations (Fig. 1a), i.e. the S13D/S16D mutations that block mHTT oligomerization and aggregation in vitro and in vivo^32,33^, and the ∆N17 mutation (a deletion of amino acid 2–16) that leads to increased oligomer formation, nuclear mHTT aggregation and HD-like disease phenotypes in vivo^29,30,34^. These two cell lines are termed mHTT-SD-GFP and mHTT-ΔN17-GFP, respectively. Similar to the mHTT-46Q-GFP cell line, both mHTT-SD-GFP and mHTT-ΔN17-GFP cell lines have neglectable aggregation at baseline and when treated with mHTT-51Q monomers (Fig. 1c,d). Interestingly, these two new cell lines showed distinct effects when treated with the mHTT fibrils and seeds. The mHTT-ΔN17-GFP cells showed a dramatic enhancement of mHTT-51Q fibrils- and seed-induced aggregation, while mHTT-SD-GFP cells showed comparable or possibly modest reduction of those induced aggregation compared to the mHTT-46Q-GFP cell line (Fig. 1c,d). Moreover, we found the mHTT-51Q seed-treated cells show more GFP^+^ aggregates compared to the mHTT-51Q fibril treated cells (Fig. 1d,e). Notably, while the size range of the aggregates is comparable between the two treatment (mostly between 0.3 and 2 μm), the mHTT-51Q seeds tend to induce a greater number of smaller-sized aggregates than the fibrils did (Fig. 1e and Supplemental Fig. S1). Together, we showed that deletion of N17 from mHTT-46Q-GFP helped to generate a highly sensitive cell line in detecting the exogenous mHTT fibrils and seeds, while maintaining minimal spontaneous aggregation of the reporter proteins at the baseline.

### Deletion of N17 domain in the mHTT-46Q-GFP reporters is necessary for enabling detection of mHTT seeding activities in HD brain samples

**Figure 2:**
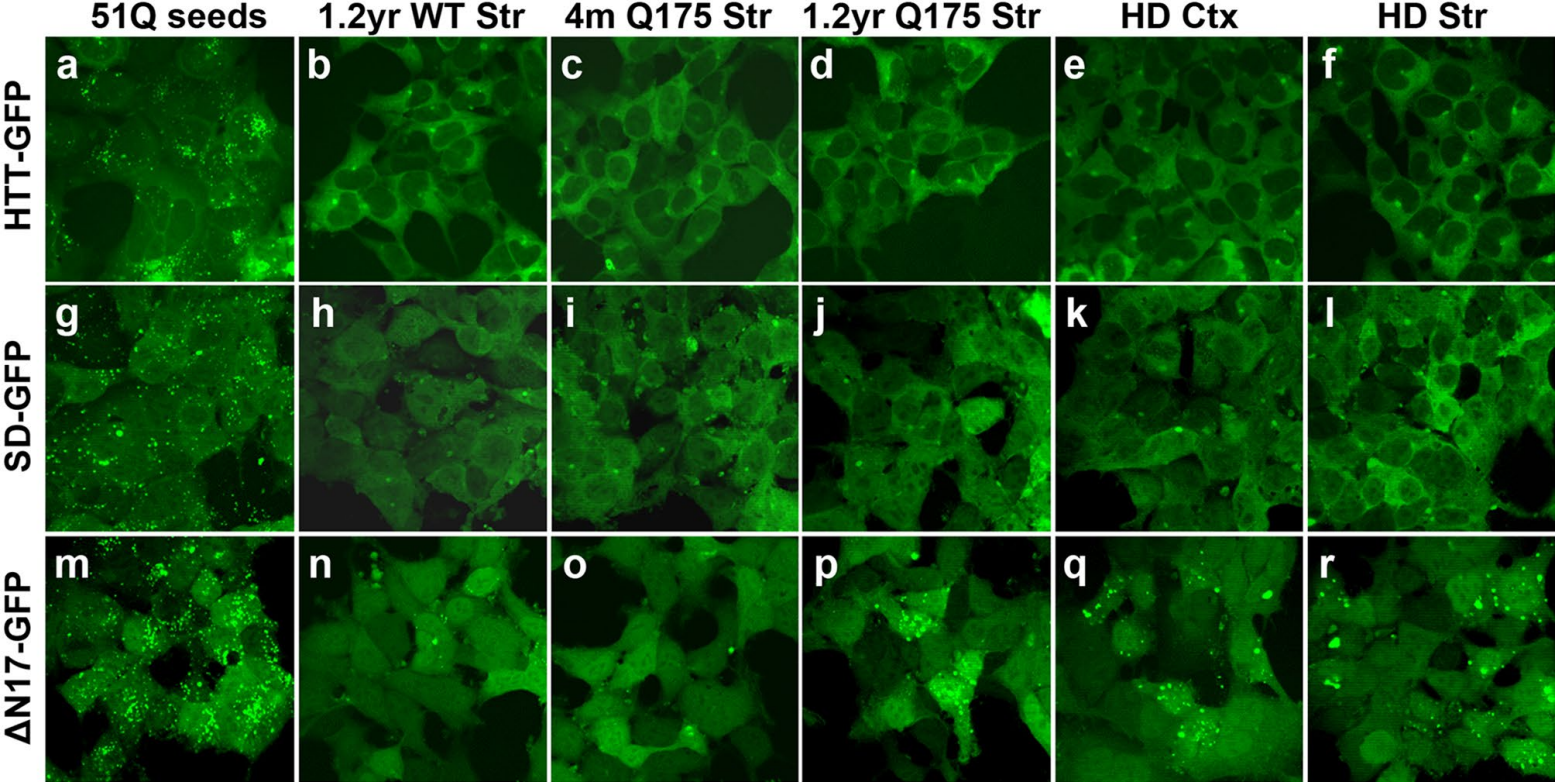
Deletion of N17 enables detection of mHTT seeding activities in HD mouse and patient brain lysates. Recombinant mHTT-51Q seeds and brain lysates from HD patients and mouse models were applied directly to mHTT-46Q-GFP (**a**–**f**), mHTT-SD-GFP (**g**–**l**) or mHTT-ΔN17-GFP cell lines (**m**–**r**) for 3 days. Representative confocal images of the cells are shown.

A litmus test for any disease protein seeding assay is whether it can reliably detect the aggregate-inducing, disease-specific protein species in the biosamples. We first tested our three reporter cell lines using brain homogenates from the Q175 mice, a full-length murine mutant Huntingtin (mHtt) knock-in mouse model carrying an expanded CAG repeat and showing early and progressive mHtt aggregation, transcriptional dysregulation, and behavioral impairment (Fig. 2)^35–38^. Among the three reporter cell lines, only the mHTT-ΔN17-GFP line is sensitive enough to show induced aggregation with sonicated striatal extracts from old (14 months of age) but not young (4 months of age) Q175 mice (Fig. 2o,p), nor with those from aged wildtype mice (Fig. 2n). Importantly, when applying sonicated cortical and striatal lysates from postmortem HD patients, we also detected GFP^+^ aggregates only in mHTT-ΔN17-GFP cells but not in the other cell lines (Fig. 2). These results demonstrate that only the reporter cells expressing the ∆N17 version of mHTT-46Q-GFP, but not the unmodified or SD versions, have the sensitivity to detect mHTT seeding activities of the brain samples. The study with Q175 striatal extracts also suggests that the seeding activities detected by the mHTT-ΔN17-GFP cells reflect specific age-dependent mHTT species emerged with disease progression, not just due to the expression of mHTT.

### Quantitative and scalable analyses of mHTT seeding species using the mHTT-ΔN17-GFP reporter cell line

**Figure 3:**
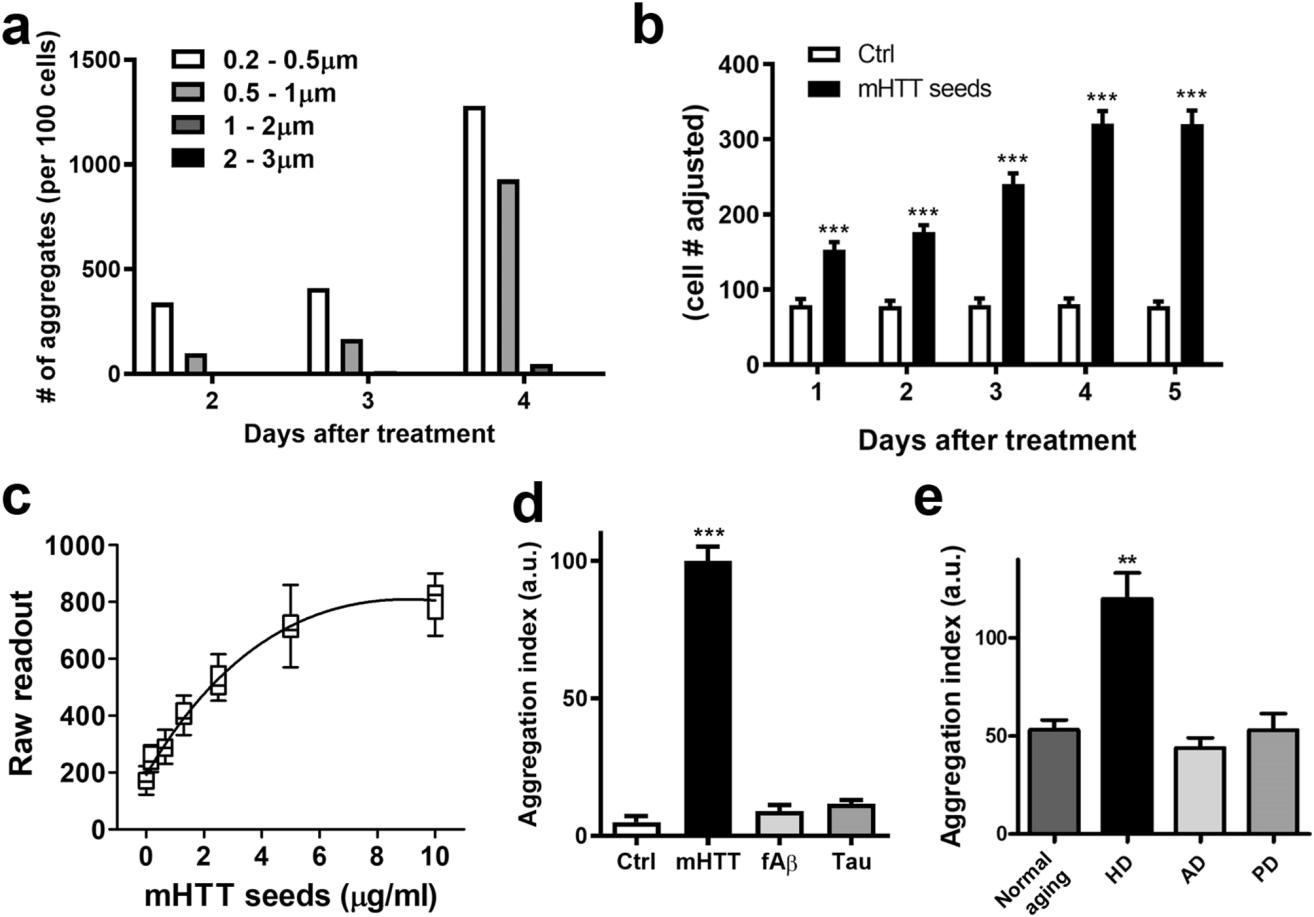
The seeding assay using mHTT-ΔN17-GFP cells is scalable and could specifically and sensitively detect mHTT seeding activity in the biosamples of HD patients. (**a**,**b**) Recombinant mHTT seeds were applied to mHTT-ΔN17-GFP cells in 24-well (**a**) and 384-well (**b**) settings for indicated duration of time (n = 6–8). (**c**) mHTT at a range of concentrations were applied to mHTT-ΔN17-GFP cells for 5 days to generate the dose–response curve (n = 6 for each condition). (**d**,**e**) mHTT-ΔN17-GFP cells were treated with sonicated recombinant protein aggregates (**d**; mHTT seed at 2 μg/ml, fAβ and pTau at 10 μg/ml) or postmortem CSF from HD, AD or PD patients (**e**) in the 384-well format for 5 days (*n* = 6–8). Confocal images were acquired manually on a Zeiss LSM510 confocal microscope and analyzed with ImageJ (**a**), or captured with automation on an ImageXpress Micro Confocal and analyzed with MetaXpress (**b**–**e**), respectively. The data are presented as mean ± s.e.m., **p < 0.01; ***p < 0.001.

To further enhance the quantification and high-throughput capability, we adapted the assay into a 384-well platform and utilized a high-content imaging system to quantify seed-induced reporter aggregation. We first compared the measurement of time-dependent aggregation using manual counting versus using automated detection after seeding (Fig. 3a,b). Both methods were capable of capturing the increase of induced GFP^+^ aggregates over time. We noticed that the smaller aggregates (< 0.9 μm) were not detected reliably by the automated image analysis, possibly due to the optical limitation and background heat noise from the instrument. Thus, for optimal signal detection over the background, we chose to only quantify the GFP^+^ aggregates at the size between 1 and 3 µm. As a result, the 384-well format and the high-content imaging system were capable of capturing the time-dependent progression of mHTT-ΔN17-GFP (referred to as mHTT-GFP thereafter) aggregation (Fig. 3b). Additionally, the level of aggregation reflected the amount of recombinant mHTT-51Q seeds applied as a function of saturation curve (Fig. 3c). Since the concentration of 2 µg/ml of extracellular mHTT-51Q seeds is well within the linear range of the dose–response curve, we used this fixed amount of mHTT-51Q seeds (2 µg/ml) as a positive control and for normalizing the aggregation readouts in all subsequent studies. We operationally defined an aggregation index of 100 arbitrary units as the number of mHTT-GFP aggregates induced by exogenous addition of 2 µg/ml of mHTT-51Q seeds onto the mHTT-ΔN17-GFP cells in the 384-well format for all later experiments.

### The mHTT-∆N17-GFP cell line is specific for detecting mHTT seeding activities originated from recombinant mHTT seeds and HD patient samples

To evaluate the specificity of the mHTT-GFP aggregation in response to the exogenous seeds, we applied sonicated fibrillar Aβ and hyperphosphorylated Tau aggregates to the mHTT-ΔN17-GFP cells. Neither of these protein aggregates induced mHTT-ΔN17-GFP aggregation in the reporter cells (Fig. 3d and Supplemental Fig. S2). We further explored whether the seeding-competent (or “seedable”) mHTT species exist in the postmortem CSF samples, i.e. the mHTT seeding activities, from HD and other neurodegenerative disease patients (UCLA’s neuropathological collection; see methods). Application of postmortem CSF from HD patients significantly increased GFP^+^ aggregates in mHTT-ΔN17-GFP cells. On the contrary, such induced aggregation was not found when the cells were treated with postmortem CSF from Alzheimer’s disease (AD) or Parkinson’s disease (PD) patients (Fig. 3e, Supplemental Fig. S2 and Table S1). Together, our results suggest that the seed-induced mHTT-GFP aggregation in mHTT-ΔN17-GFP cells is highly specific to recombinant mHTT aggregates, and to the seeding-competent species present in the CSF of HD patients.

### The mHTT seeding activities in the postmortem CSF of the HD patients are correlated with neuropathological grades of the brains

**Figure 4:**
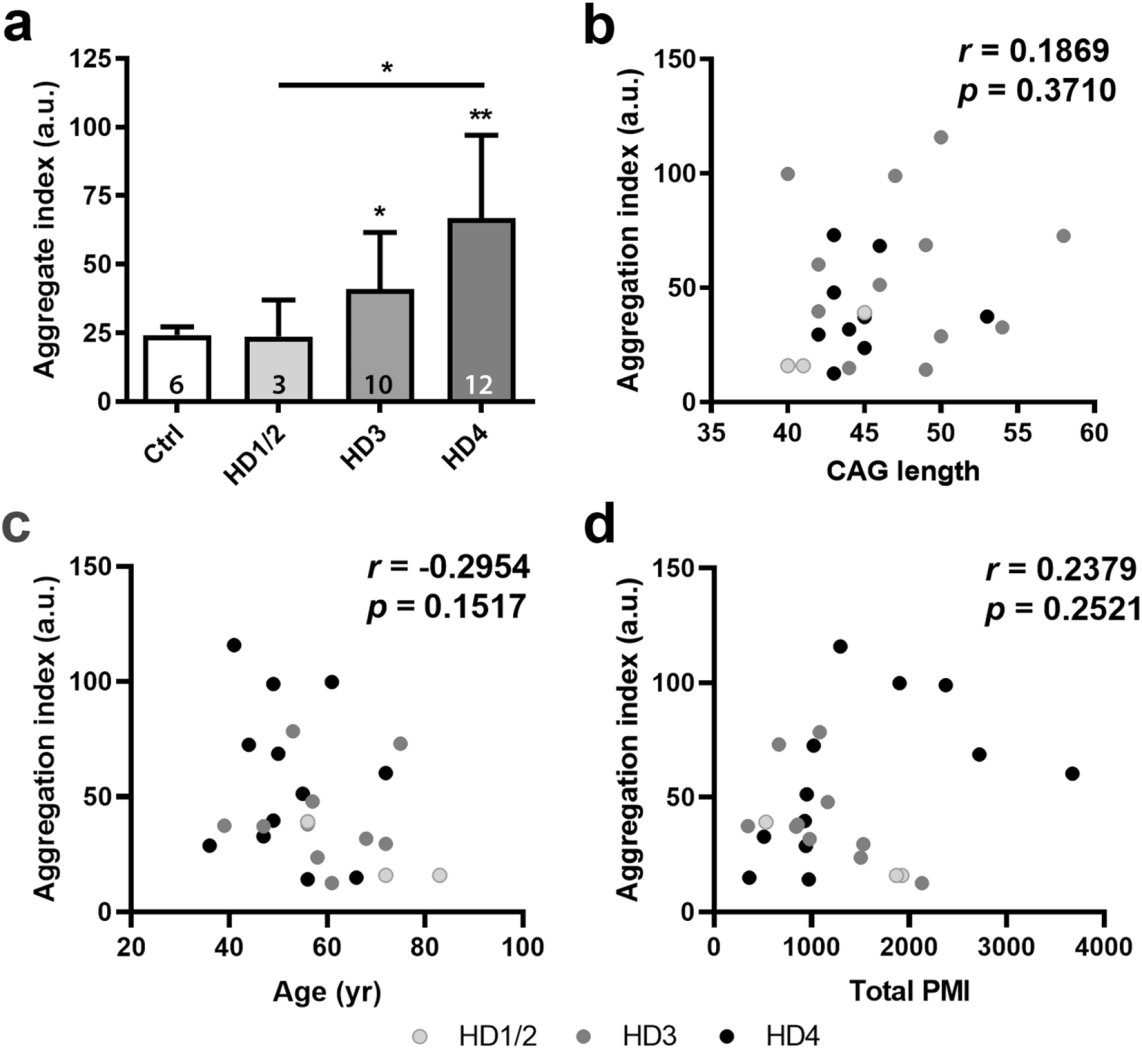
The mHTT seeding activities in the postmortem CSF is correlated with neuropathological grade based on striatal degeneration. The disease status of the patients were graded based on their neuropathological features. Postmortem CSF samples from these subjects and non-HD controls were applied to the mHTT-ΔN17-GFP cells at 1:20 dilution in the 384-well format for 5 days. Confocal images were acquired on ImageXpress Micro Confocal and used to measure induced mHTT-GFP aggregation using MetaXpress. (**a**) CSF samples were grouped based on their pathological grading. The number of samples is as shown in the bar. HD1 and HD2 samples were grouped together for statistical analysis. One-way ANOVA with Tukey post-hoc analysis was performed to determine the *p* value versus the non-HD controls, or otherwise as indicated. Bar graphs are shown as mean ± s.d. *p < 0.05, **p < 0.01. (**b**–**d**) Correlation analysis between the CSF seeding activities and the CAG repeat length (**b**), the age of the HD patients (**c**) or PMI of the CSF samples (**d**). Individual dots are color coded by their neuropathological scores. Pearson’s correlation analysis was performed to determine the Pearson’s *r* and *p* value.

We next investigated whether the levels of CSF mHTT seeding activities correlate with the pathological progression in the brain, which was measured by Vonsattel neuropathological grades, a postmortem evaluation of neuronal cell loss in the caudate and putamen of HD patients^39^. We assayed a panel of autopsy CSF samples with HD neuropathological staging performed by Dr. Vonsattel at Columbia University Medical Center (Supplemental Table S2). The autopsy CSF from HD Stage 3 and Stage 4 subjects (NY Brain Bank or NYBB cohort) induced significantly more mHTT-GFP aggregation compared to controls, while CSF from HD1 and HD2 patients did not significantly increase aggregation in these cells (Fig. 4a). The statistical significance of the latter groups may be in part limited by the small sample size (*n* = 3 for HD1 and HD2 combined) (Fig. 4a). Importantly, CSF from HD4 subjects also induced significantly more mHTT-GFP aggregation than those from HD1 and HD2 patients, suggesting an HD neuropathological stage-dependent increase of seeding-competent mHTT species in the CSF. To evaluate possible confounding factors, we tested linear models of the aggregation index regressed on HD grade (treated as a categorical variable) and potential confounders including age, PMI and gender (Supplemental Table S4). We found that the association between HD4 and the aggregation index remained significant (*p* = 0.02). We further test the all combination of these covariates plus CAG length. HD grades regressed on the aggregation index remains the best model, since addition of other covariates was more likely deteriorate the fit (Supplemental Table S4). Analyzing the confounders individually, the age and CAG length of subjects are not correlated with the seedability of HD CSF (Fig. 4b,c). We also found no correlation between PMI and the seeding activities of the CSF (Fig. 4d). Thus, the strong correlation between the level of seeding-competent mHTT species in CSF and HD pathology suggests that these mHTT species in CSF, which could be detected by the mHTT-ΔN17-GFP reporter cell assay, might elevate as neuropathology progressing in the brain.

### Preliminary analysis of mHTT seeding activities in CSF of living HD patients suggests its association with disease progression

**Figure 5:**
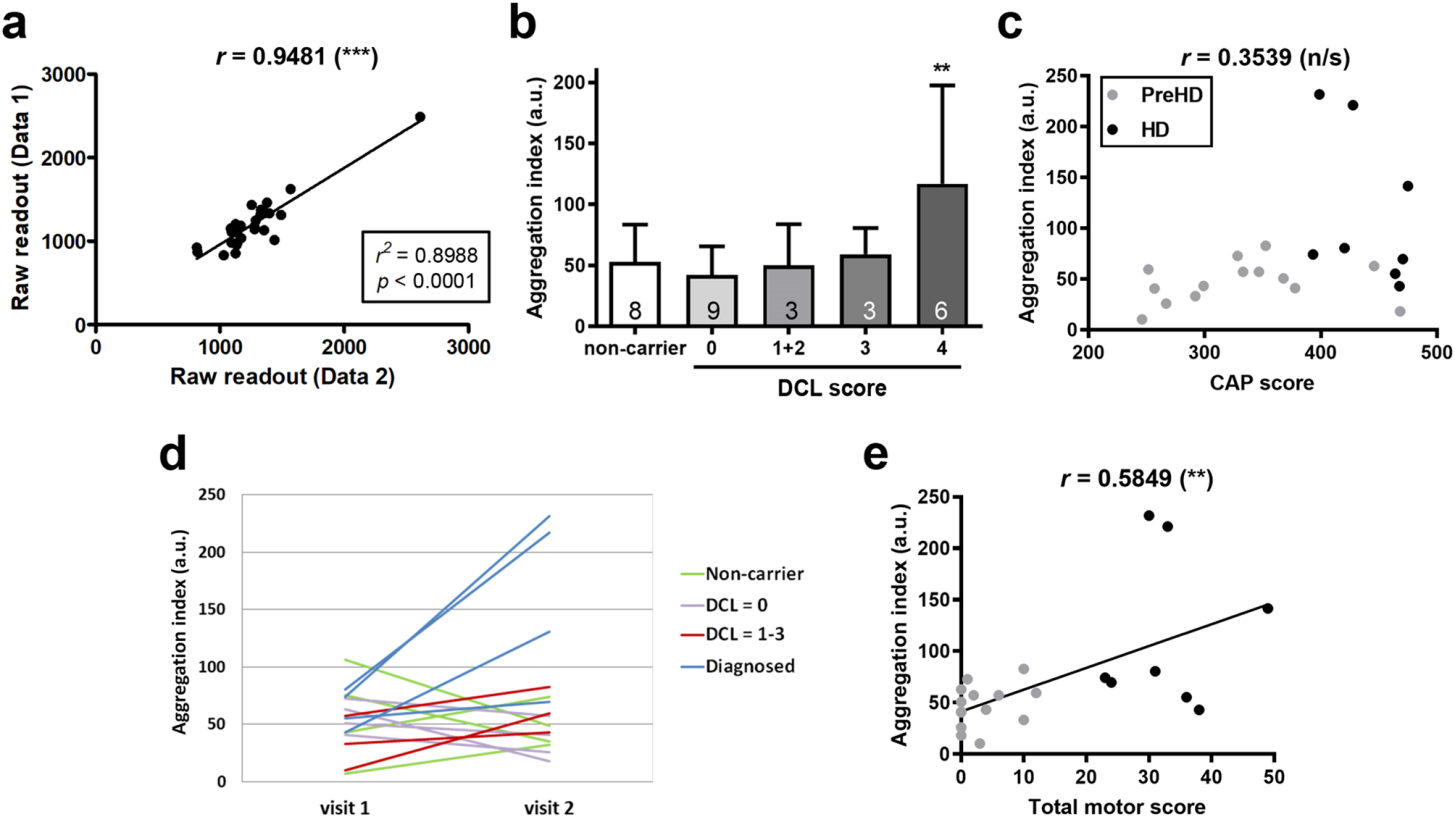
High reproducibility of detecting disease-related mHTT seeding activities in CSF from living HD patients. (**a**) Identity blinded CSF samples from the PREDICT-HD collection were provided by BioSEND and applied to the mHTT-ΔN17-GFP cells at 1:20 dilution in the 384-well format for 5 days. Confocal images were acquired with automation and batch analyzed for aggregation scores. Upon unblinding the samples, the two aggregation readouts from the duplicated aliquots of the same CSF samples were plotted and showed high reproducibility between the duplicates of the samples using this assay. (**b**) The HD patients, mutation carriers and non-carrier controls were grouped based on their DCL scores. DCL1 and DCL2 are grouped together due to their small sample sizes. Numbers in the bars indicate the *n* of each group. (**c**) The correlation analysis of the CSF seeding activities and the CAP score. (**d**) The measured aggregation indices of both visits from the same individuals were plotted to visualize the change of CSF ceding activity. (**e**) The total motor scores was plotted against the CSF mHTT seeding activity to analyze their association. Pearson’s correlation coefficients (*r*) for (**c**) and (**e**) were calculated and noted on the top of the plots. Statistical significance (*p* value) of the correlation is shown in parentheses. Additional linear regression analyses were performed for (**a**,**e**), and shown in the squares. Bar graphs are presented as mean ± s.d. **p < 0.01; n/s = not significant.

Postmortem pathological grades indicate the end stage condition of the brain. However, whether the pathological measurement could be related to clinical severity of HD remains debatable. We began to address this question by investigating whether our assay could detect mHTT seeding species in the CSF from living HD gene carriers and patients, and if such study could provide initial insights on the role of seeding activities with the disease. The CSF samples were acquired from the PREDICT-HD collection (NINDS BioSEND), a longitudinal phenotypic and biomarker study of prodromal HD (see “Methods” section and Supplemental Table S3)^40^. In PREDICT-HD, the enrolled individuals were evaluated annually for motor and cognitive functions, performed brain imaging and collected biosamples. They are categorized by the diagnostic confidence level (DCL), where DCL0 means indistinguishable from healthy individuals, DCL2 and DCL3 are prodromal stages with increasing likelihood of HD diagnosis, and DCL4 is most likely to be clinically diagnosed HD. To prevent experimental bias, we were provided 60 PREDICT-CSF samples by NINDS/BioSEND and performed the seeding assays while blinded to any identification or clinical information. Samples were unblinded only after the results were submitted back to NINDS BioSEND. We found that the 60 CSF samples were actually duplicates of 30 samples collected from 2 visits of 15 individuals (including controls, mHTT gene carriers and HD patients; Supplemental Table S3). This experimental design was aimed to evaluate incurred sample reproducibility of our assay. The results demonstrated satisfying high incurred sample reproducibility (Pearson’s *r* = 0.9481, *p* < 0.0001; Fig. 5a). We found a significant increase in the mHTT seeding activities of the CSF only from the DCL4 group, but not from the pre-HD (DCL1–DCL3) subjects, even though they already displayed some signs of motor abnormalities (Fig. 5b). To account for correlation of aggregation index measurements of the two visits for each subject, we used linear mixed model with the variable of interest (DCL score) as a categorical fixed effect and the subject as a random effect. Here, non-carrier subjects and the DCL0 HD carriers were combined as the control group of healthy individuals to improve statistical power. We found significant association for DCL4 versus DCL0 (healthy individual group) in the pairwise comparison and for DCL4 versus the rest groups in the full model (Supplemental Table S5). To ensure that this result is not due to confounding, we carried out a comprehensive study of regression with all combination of the following 8 covariates: age, sex, education years, height and BMI, CAG (high), CAG (low) and CAP score (there are 2^8^ = 256 combinations). When the covariates are added individually, the association between aggregation score and DCL4 remained significant (Supplemental Table S6). None of the covariates is significantly (*p* < 0.05) associated with aggregation index in any of the models. There are combinations of covariates (e.g., all 8) with which the association with DCL becomes non-significant, likely because these combinations of covariates explain a large fraction (mostly over 50%) of the DCL score variation. As a diagnostic check, especially for the small PREDICT-HD sample size, we tested normality of the residuals of aggregation index using Shapiro–Wilks test and found non-significant results (Supplemental Table S7).

Another interesting finding is the lack of significant correlation between mHTT seeding activities and the CAG-age product (CAP) score (Pearson’s *r* = 0.3539; Fig. 5c), a mathematical predictor of HD progression^41^, and CAG repeat length (Pearson’s *r* = 0.09349; Supplementary Fig. 3b). We also found no significant associations with CAP and CAG using linear mixed effects models (Supplemental Table S5). This finding suggests that our seeding assay is not tracking the CAP score, and also differentiates our seeding assay from the clinically used SMC assay, which is highly correlated with the CAP score^10^. Notwithstanding the need to include additional samples to further test the robustness of our seeding assay in clinical samples, our findings thus far suggest that the mHTT seeding activities may be significantly increased upon HD diagnosis and potentially track aspects of the disease that are not measured by the traditional CAP scores or SMC assay.

Further analysis focused on the comparison of the CSF samples collected from two visits, 1 year apart, of the same individuals. This analysis should be considered preliminary due to the limited number of multi-visit CSF samples available from PREDICT-HD. Despite such limitation, we found a significant increase in the change of the CSF seeding activities (∆ aggregation index) in individuals diagnosed with HD but not in non-carriers or HD gene carriers in the DCL0 to DCL3 categories (Fig. 5d and Supplemental Fig. S3a). These preliminary results suggest that the level of mHTT seeding species in CSF is significantly progressive only after the subjects are diagnosed.

We next performed a correlation analysis of the aggregation index with a series of clinical scores, assessed at the time of visits. Again, while these analyses should be considered preliminary, we found a significant correlation between the CSF seeding activity and the disease severity. Increasing seeding propensity of the CSF was significantly correlated with the UHDRS total motor score (Pearson’s *r* = 0.5849, *p* = 0.0054; Fig. 5e) and total function capacity (TFC; Pearson’s *r* = − 0.7476, *p* < 0.0001; Supplemental Fig. S3 and Table S3)^41^. We again used mixed effect models to test these associations while accounting for repeat visits and found that the associations of aggregation index and TFC remain significant even after Bonferroni correction for the 69 tested clinical variables (Supplemental Table S7). In contrast, we did not find that the CSF seeding propensity significantly correlate with cognitive tests (e.g. Stroop color naming and word reading scores), while some of these scores are significantly higher in PreHD than manifest HD subjects, nor with the volume changes in cortical and striatal brain regions (Supplemental Fig. S3 and Table S3 and S8).

Together, our study suggests that the mHTT seeding activities in CSF appear to significantly increase as HD diagnosis and are significantly correlated with clinical severity as the disease progress in the subjects. A limitation of our current study is the relatively small number of longitudinal CSF samples available from manifest HD patients with clinical data, and therefore it should be considered preliminary findings. Future longitudinal studies with a large cohort of premanifest and manifest HD patients will be needed to rigorously evaluate the relationships between CSF mHTT seeding activities and disease onset and progression in HD.

### Blocking HD CSF mHTT seeding activities with chaperone DNAJB6 and antibodies against the polyproline domain of HTT

We next sought to apply our cell-based mHTT seeding assay to evaluate genes and molecules that block the pathological seeding activities in HD patient CSF, and hence to gain insights into the molecular bases of such disease-related mHTT seeding activities.

**Figure 6:**
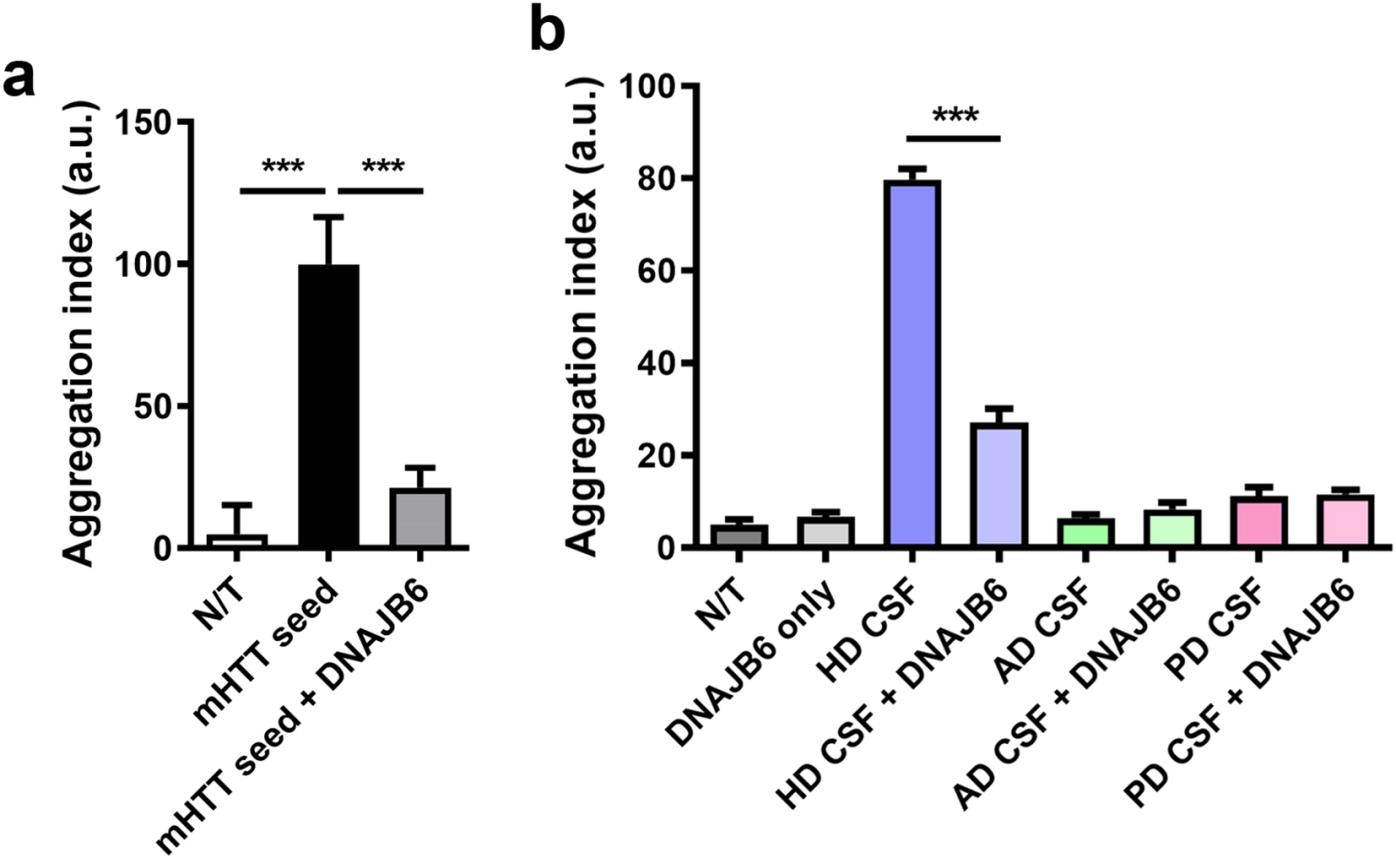
Chaperone DNAJB6 blocks the seeding activities of HD patient CSF. The mHTT-ΔN17-GFP cells were transfected with DNAJB6 1 day prior to the application of mHTT51Q seeds (2 µg/ml, **a**) or postmortem CSF from HD, AD and PD patients (1:20, **b**). The assays were performed in the 384-well format. Confocal images were acquired with automation 5 days after seeding and batch analyzed to calculate Aggregation indices of the samples. Bar graphs are presented as mean ± s.e.m., *n* = 6–8, ***p < 0.001, N/T = non-treated controls.

DNAJB6 is a member of the Hsp40 chaperone protein family and has been shown to delay polyglutamine aggregation through inhibition of early nucleation and the subsequent amyloid fibril formation^42,43^. We investigated if recombinant seed- or patient CSF-mediated induction of mHTT-GFP aggregation could be modified by elevated DNAJB6 expression^43^. We found that DNAJB6 overexpression almost completely abolished the recombinant mHTT seed-induced aggregation (Fig. 6a). Similarly, we observed a significant DNAJB6-mediated suppression of mHTT-GFP aggregation induced by the HD patient CSF (Fig. 6b). However, no difference is observed in the groups treated with CSF from AD and PD patients with or without DNAJB6 overexpression (Fig. 6b). The results not only further confirmed that AD and PD CSF only induce neglectable mHTT seeding activities, if any, in our cellular assay, but also showed that such baseline seeding activities are not template-dependent and thus not sensitive to elevated DNAJB6 levels. Together, our findings suggest that induced aggregation by HD CSF is likely a DNAJB6-sensitive, mHTT seed-mediated nucleation and fibrillation process.

**Figure 7:**
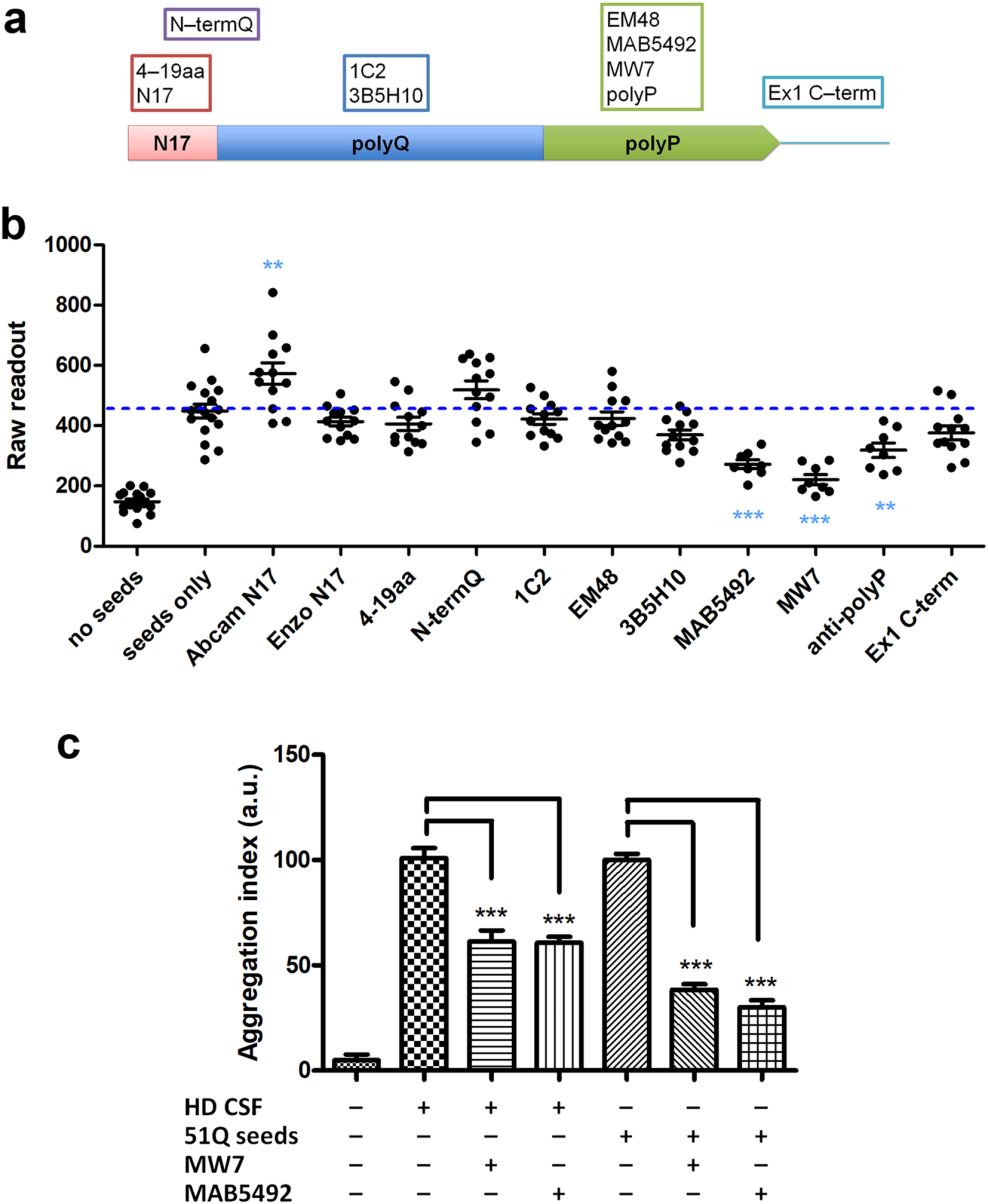
Only anti-polyP antibodies were capable of blocking the seeding activities of HD patient CSF. (**a**) An illustration indicates the epitopes for the HTT antibodies. (**b**) The mHTT-51Q seeds were preincubated with the indicated antibodies before administration to mHTT-ΔN17-GFP cells in the 384-well format. The epitopes of the antibodies are indicated in the upper panel. Confocal images were acquired with automation 5 days after seeding and batch analyzed. Statistic comparisons were made between the antibody-treated groups with the mHTT-51Q seed-only group. *n* = 8–12. (**c**) The mHTT-51Q seeds and postmortem CSF from HD patients were immunodepleted with two anti-polyP antibodies (as indicated) overnight and applied to mHTT-ΔN17-GFP cells for 5 days. *n* = 6. Statistic results are presented as mean ± s.e.m. **p < 0.01; ***p < 0.001.

The utilization of epitope-specific antibodies to block protein–protein interaction or to deplete mutant proteins from the biosamples is a well-established strategy to evaluate the molecular nature of pathological seeding activities, and has been shown to effectively inhibit the intracellular aggregation and propagation of the disease proteins, e.g. αSyn and Tau, by extracellularly administered antibodies^44,45^. To investigate which mHTT-exon 1 domains may be critical in mediating the template-based seeding activities described above, we tested a battery of antibodies against different domains of mHTT-exon 1 (Fig. 7a). Three out of four antibodies against the polyproline-rich domain (polyP) significantly reduced mHTT-GFP aggregation induced by the recombinant mHTT seeds (Fig. 7b). In contrast, none of antibodies against the N17 domain or the polyQ domain could block the mHTT seeding activity (Fig. 7b). In fact, one of the three anti-N17 antibodies (i.e. Abcam anti-N17) even slightly but significantly enhanced the nucleation. Although the blocking efficiency may vary among antibodies against same epitopes, the findings suggest that the polyP domain is critical for recombinant mHTT seeds to elicit template-based aggregation of mHTT-∆N17-GFP intracellularly.

We next tested whether the CSF mHTT seeding species could be immunodepleted by anti-polyP antibodies to reduce their seeding propensity. Two anti-polyP antibodies (MW7 and MAB5492) were used to immunodeplete mHTT seeding species from the HD CSF samples. The immunodepleted CSF samples were then applied to the mHTT-ΔN17-GFP cells. We found that HD patient CSF, immunodepleted by both anti-polyP antibodies, significantly diminished its seeding ability in the assay (Fig. 7c). This result strongly supports that the seeding activities presenting in the HD patient CSF samples are consisted of HTT species with an exposed polyP domain, and this domain is necessary to mediate the seed-induced aggregation in the reporter cells.

## Discussion

To specifically detect mHTT seeding species, especially those in HD patient CSF, we developed a sensitive reporter cell assay with high-throughput capability. Two key genetic engineering of the reporter protein are the use of moderate CAG repeats (46Q) for minimal aggregation at the baseline and deletion of the N17 domain, which showed remarkable enhancement in sensitivity for detecting mHTT seeding species, including those in HD patient biosamples. Furthermore, by adapting the new cell line into a 384-well, high-content imaging assay platform, we are able to reproducibly detect and quantify mHTT seeding activities in the HD patient CSF. Our preliminary study of clinical samples reveals a significant increase in the mHTT seeding activities in the CSF of HD gene carriers upon their initial diagnosis of HD (i.e. DCL4), and a continuing increase of such seeding activities with progression of neuropathological grades in the postmortem samples. Finally, our assay reveals the mHTT seeding activities could be inhibited by overexpression of the chaperone DNAJB6 and by immunodepletion using antibodies against the polyP domain of mHTT. Further studies of larger premanifest and manifest HD patient cohort are needed to fully evaluate the utility of longitudinal CSF mHTT seeding activities, as detected by the highly sensitive cell-based assay, in tracking the clinical onset and progression of HD. Finally, our study also demonstrated the utility of this cell-based assay to evaluate molecules that can suppress the mHTT seeding activities from HD patient CSF, which in turn could be further investigated for their roles in disease modifications.

One important finding of this study is that the propensity of the mHTT seeding is relatively minimal in HD gene carriers prior to the symptom onset, and is significantly elevated upon disease diagnosis and appears to continuously increase with neuropathological progression. Previous studies have reported increased mHTT species in the CSF before and after the disease onset^9,10,26,46^. However, changes in the levels of mHTT seeding species have not been described over the course of the disease and with the concurrence of symptoms. In this study, we demonstrate the neuropathological and clinical stage-dependent increase (i.e. with HD grades in postmortem CSFs and with DCL scores in the PREDIT-HD cohort, respectively) in mHTT CSF seeding activities. Despite the limited availability of multi-visit samples in PREDICT-HD, our study also showed that the seeding propensity of CSF is also augmented in subsequent visits of HD patients but not in those of premanifest mutant carriers. Moreover, our preliminary findings showed the CSF seeding propensity is positively correlated with the disease severity, including TFC and total motor score, similar to the currently clinically used SMC assay^10,11^. The SMC assay is significantly correlated with the CAP score^10,41^. One important question is whether the CSF mHTT seeding activities are tightly correlated with the CAG length and/or age of the patients. We found that the CSF mHTT seeding propensity does not appear to correlate with the CAP score of the patients. Thus, our assay does not seem to probe the same mHTT species in the patient CSF as the SMC assay. We acknowledge that a limitation of our study is the relatively small number of CSF samples from premanifest and manifest HD patients used. Therefore, our findings related to the relationships of CSF seeding activities and clinical disease stages should be considered preliminary. Future large-scale studies with longitudinal CSF samples and clinical data from both HD gene carriers and patients is needed to rigorously evaluate the clinical utility of this high-throughput, cell-based mHTT seeding assay in monitoring HD onset and progression and in evaluating efficacies of HD therapeutics.

We demonstrated that overexpression of DNAJB6 in the reporter cell line can block the seeding activities from preformed seeds and patient CSF. These findings confirmed that our assay is detecting template-based, aggregate-inducing activities of mHTT species in the CSF. It further suggests that studying chaperone-mediated mHTT proteostasis, such as those mediated by DNAJB6 and DNAJB8^42,43^ and other chaperones^47^, could be a meaningful approach to identify molecular targets and candidate therapeutic reagents to block mHTT seeding in the patients.

A key advance in our study is to provide strong evidence that the CSF seeding activities from HD patients are likely to be mediated by specific mHTT species, of which the polyP region is exposed and might play a key role in the seeding process. We showed that antibody-blockade and immunodepletion with three different anti-polyP antibodies could significantly reduce mHTT-GFP aggregation induced by recombinant seed and CSF from HD patients, while anti-N17 or anti-polyQ antibodies could not elicit similar effects. This result is consistent with prior studies showing that co-expressing polyP-specific intrabodies inhibited aggregation of mHTT proteins in HD transgenic mice^48–50^. It is worth noting that a commonly used anti-HTT antibody, EM48, of which epitope is mapped to the polyP region, could not elicit the same blocking effect. This might be attributed to the intrinsic difference of antibodies in epitope recognition and affinity. Recent structure studies suggest a “bottle brush” model for HTT exon 1 structure, with the polyP domain forms bristled helix-rich structures projecting away from the fibril core of N17 and polyQ^51,52^. Aggregation of mHTT is a stepwise event, initiated by N17 interaction, followed by β-sheet formation of polyQ and, lastly, crowding of the polyP bristles. Given the slowest step lies on the final structure maturation as polyP crowding^52^, presence of antibodies against the polyP domain might reduce mHTT-GFP aggregation through interference with polyP bristles compaction. Future studies are needed to test whether extracellular delivery of anti-HTT-polyP antibodies could be an effective measure to block the propagation of mHTT aggregation and HD pathogenesis in vivo.

Finally, our scalable, highly sensitive cell-based mHTT seeding assay can be used in large scale perturbation studies, e.g. anti-HTT antibodies, intracellular gene-based screenings (i.e. shRNA, CRISPR/Cas9, or cDNA overexpression) and small molecule screenings to systematically identify molecules that could modulate mHTT seeding activities. A good example is the antibody screening reported in this paper. This is made possible as our assay requires only very small amount of CSF (i.e. 10–20 μl) per sample. Thus, future large-scale, phenotype-driven screening with our assay could identify genetic targets or small molecules to curb such pathogenic activities in the patients.

In conclusion, we reported the development of a novel high-throughput cell-based system that could sensitively and specifically detect disease-related mHTT seeding activities in HD patient CSF. By using this platform, we identified molecules (i.e. DNAJB6 expression or anti-HTT polyP antibodies) that could block such seeding activities. Our study will enable future systematic analyses of the pathogenic mHTT seeding activities in HD patient CSF to evaluate its potential utility as disease-progression biomarkers, and for large-scale screening of molecules to ameliorate pathogenic seeding activities in HD.

## Methods

### Human brain and CSF samples

Prior to the seeding assays, samples were randomized and blinded to investigators. Autopsy brain tissues of subjects with HD were obtained from UCLA Human Brain and Spinal Fluid Resource Center (Supplemental Table S1). Postmortem CSF was provided by the New York Brain Bank at Columbia University. Vonsattel neuropathological grades of the HD patients was determined by the New York Brain Bank using their respective brain tissues (Supplemental Table S2). PREDICT CSF samples were obtained from BioSEND at Indiana University. Correlated clinical information and scores were provided by Dr. Jane Paulsen at University of Iowa via dbGaP (Supplemental Table S3)^40^. The PREDICT-HD participants visits the centers annually for biosample collection (e.g. blood, CSF and saliva), brain imaging and cognitive and other neurological evaluation^53,54^. CSF were collected through lumbar puncture (LP) after fasting. Basic CSF quality analyses were conducted for cell count, erythrocytes, total protein, and glucose^54^. All paired multi-visit CSF samples from the same individuals were collected 1 year apart.

### Animals

Heterozygous Q175 mHtt knock-in mice carrying mHtt with about 190 CAG repeats were obtain from Jackson Laboratory and aged to designated ages. Animals were housed in standard mouse cages under conventional laboratory conditions, with constant temperature and humidity, 12 h/12 h light/dark cycle and food and water ad libitum. All animal studies were carried out in strict accordance with National Institutes of Health guidelines and approved by the UCLA Institutional Animal Care and Use Committees. The mice were anesthetized by pentobarbital. Brains were removed and carefully dissected to obtain cortical and striatal tissues. Dissected tissues were snap frozen in dry ice and stored in − 80 °C before further processing.

### Antibodies

Anti-HTT antibodies EM48, 1C2, 3B5H10 and MAB5492 were obtained from Millipore. An anti-HTT antibody MW7 were from the Developmental Studies Hybridoma Bank. Two anti-N17 antibody were purchased from Abcam and Enzo. Anti-N-termQ, anti-4-19aa, anti-polyP and anti-Ex1 C-term of mHTT antibodies were provided by CHDI foundation.

### Brain lysate preparation

Cortical and striatal samples of HD patients or mice were submerged in PBS and homogenized using a Dounce homogenizer. Homogenates were then sonicated for two 10 s-on/10 s-off cycles and centrifuged at 3000×*g* for 10 min to remove undissolved tissue debris. Supernatants were re-sonicated and stored at − 80 °C until use.

### Generation of cell lines and cell culture

DNA constructs of mHTT exon 1 (46Q) fragment C-terminally tagged with EGFP were cloned into a mammalian expression vector, pcDNA3.1, to generate the mHTT-46Q-GFP plasmid. mHTT-SD-GFP and mHTT-ΔN17-GFP plasmids were generated by direct point mutation of S13/S16 residues to D13/D16 and deletion of 2–16 a.a. residues of mHTT on the mHTT-46Q-GFP plasmid using a QuikChange site-directed mutagenesis kit (Agilent), respectively. Resulting constructs were validated by Sanger’s sequencing. For generation of stable reporter cell lines, HEK293 cells (ATCC CRL-1573, Manassas, VA) were transfected with one of aforementioned plasmids using FuGENE HD (Promega) as described by the manufacturer's protocol. Two days following transfection, the culture media was replenished and G418 (500 ng/ml) was administered, allowing the selective propagation of transfected cells in culture. Neomycin resistant, fluorescent-labeled colonies were identified by a fluorescent microscope on day 14. Single transgenic colonies were picked by a micropipette, and transferred into a 24-well culture dish. The cells continuously proliferated in the presence of G418 (reduced to 200 ng/ml), and the expression of transgenes was monitored by a fluorescent microscope. Cellular expression of mHTT-GFP was confirmed by immunostaining using an anti-HTT antibody, EM48 (Millipore). Stable cells were maintained in Dulbecco’s modified Eagle’s medium (DMEM; ThermoFisher) supplemented with 10% fetal bovine serum (FBS) and gentamycin (25 ng/ml) at 37 °C.

### Generation of recombinant proteins and seeds

Recombinant mHTT-51Q proteins were produced as previously described^30^. In brief, Rosetta 2 (DE3) pLysS competent cells expressing pGEX-mHtt-Ex1-Q51 plasmid were induced with 1 mM IPTG for 2.5 h at 16 °C. Cell pellets were lysed using an Emulsiflex (Avestin, Ottowa, Canada) and incubated with GSH-Sepharose resin (GE Healthcare, Pittsburgh, PA, USA) and washed with 0.1% Triton, 500 mM NaCl, and 5 mM Mg-ATP before eluting protein with 15 mM Glutathione. Protein was concentrated and buffer exchanged with 50 mM Tris-HCl, pH 8.0; 100 mM NaCl; 5% glycerol. Concentrated mHTT-51Q protein was 0.2 μm PVDF filtered. Aggregation reaction was conducted by incubating 20 μM of mHTT-51Q in TEV reaction buffer (ThermoFisher) at 30 °C for 60 h. Aggregates were centrifuged at 16,000×*g* for 1 h at room temperature to pellet fibers, which were resuspended in TEV buffer and then sonicated 1 s-on/1 s-off pulsing using a Fisher Scientific 120W Sonic Dismembrator (ThermoFisher). The size of the sonicated fibers was measured by DLS using a Zetasizer Nano ZS (Malvern, Worcestershire, UK).

Aβ peptides corresponding to the human Aβ amino acids 1–42 were purchased from American Peptide Co. (Sunnyvale, CA) and dissolved to a final concentration of 1 mg/ml in DMSO. To generate fAβ seeds, lyophilized Aβ peptides were reconstituted in sterile distilled water and incubated at 37 °C for 1 week to allow fibrillization. fAβ was then sonicated for two 10 s-on/10 s-off cycles to generate fAβ seeds.

### Cell-based seeding assay

One day prior to seeding, cells were plated on poly-d-lysine coated coverslips or 384-well plates and cultured with DMEM plus 10% FBS. mHTT-51Q (2 μg/ml or designated concentrations), Aβ (10 μg/ml), Tau (10 μg/ml), brain extract (10 μg/ml of total protein) or CSF (at final 1:20 or other designated dilutions) were directly applied to the cells. For DNAJB6 experiments, cells were transfected with a DNAJB6 expressing plasmid pcDNA5/FRT/TO DNAJB6b^43^ a day prior to seeding. The media were then replenished and recombinant mHTT seeds (2 μg/ml) or CSF (1:20; UCLA in-house collection) were added. For evaluating the role of the mHTT domains in seed-induced aggregation, two methods were used to apply mHTT domain-specific antibodies: (1) immunoblocking—the recombinant seeds were preincubated with antibodies (2 μg/ml) for 10 min before applying to the cells; (2) immunodepletion—recombinant mHTT seeds or CSF (UCLA’s neuropathological collection) were incubated with 2 μg of antibodies and 10 μl of Protein A/G agarose beads in 100 μl of PBS overnight. The mixture were then centrifuged at 13,000×*g* for 2 min. The resulting supernatant were applied to the cells at the concentration as if they had not been immunodepleted (seeds at 2 μg/ml and CSF at 1:20). The cells were then incubated for 3 or 5 days to allow aggregate formation for low throughput (i.e. coverslips) or high throughput (i.e. 384-wells) experiments, respectively. Confocal images were taken using a Zeiss LSM 500 confocal or ImageXpress Micro Confocal (Molecular Devices) and analyzed by ImageJ or MetaXpress (Molecular Devices), respectively. The particle measurement function in ImageJ were used to quantify the aggregates in the cells. High-content image analysis is described below.

### High-content imaging and analysis

High-content images were acquired using automated the ImageXpress Micro Confocal system with a 10 × objective at the confocal slit mode. Z-stacked images were taken to ensure capturing all aggregates within the cells. Four sites per well were imaged to avoid in-well variation. Maximum intensity projected images were analyzed by MetaXpress with Granularity module at Standard analysis mode. Raw readouts were normalized to the internal controls within each plate. The normalized values are labeled as “aggregation index” in the figures. For all experiments, 6–8 wells were used as the technical replica and averaged to generate one data point for a biological replicate.

### Immunodepletion of mHTT seeding species from CSF

HD patient CSF (1:10) were mixed with 2 μg of an anti-HTT antibody and Protein A/G-conjugated agarose beads (1:10, ThermoFisher) in DMEM and incubated with rocking for 24 h at 4 °C. The mixture were centrifuged at 13,000×*g* for 2 min. The supernatants were then immediately applied to the cells and incubated for designated time for the seeding assay.

### Statistical analyses

Group comparisons without covariates were performed using one-way ANOVA with Tukey post-hoc test. To assess possible confounders, linear models were used for post-mortem CSF data, while linear mixed effect models were used for PREDICT-HD data to account for the fact that there are two data points (visits 1 year apart) for each subject. Bonferroni correction was used to account for testing of 82 clinical scores. Shapiro–Wilks test was used to test for normality of residuals. Pearson correlation and the corresponding significance estimate were used to quantify association between continuous variables.

## Supplementary information

Supplementary Information.

Supplementary Table S4.

Supplementary Table S5.

Supplementary Table S6.

Supplementary Table S7.

Supplementary Table S8.
