# Supplementary material for: Disease-related Huntingtin seeding activities in cerebrospinal fluids of Huntington’s disease patients
Source: Sci Rep. 2020 Nov 20;10:20295. doi: 10.1038/s41598-020-77164-1 (PMC7679413; doi:10.1038/s41598-020-77164-1)
Supplement: Supplementary file 1 — Supplementary Information. [file 41598_2020_77164_MOESM1_ESM.docx]

**Supplementary Figures**

**Disease-Related Huntingtin Seeding Activities in Cerebrospinal Fluids of Huntington’s Disease Patients**

**C.Y. Daniel Lee, Nan Wang, Koning Shen, Matthew Stricos, Peter Langfelder, Kristina H. Cheon, Etty P. Cortés, Harry H. Vinters, Jean-Paul Vonsattel, Nancy S. Wexler, Robert Damoiseaux, Judith Frydman and X. William Yang**

**FIGURE S1**


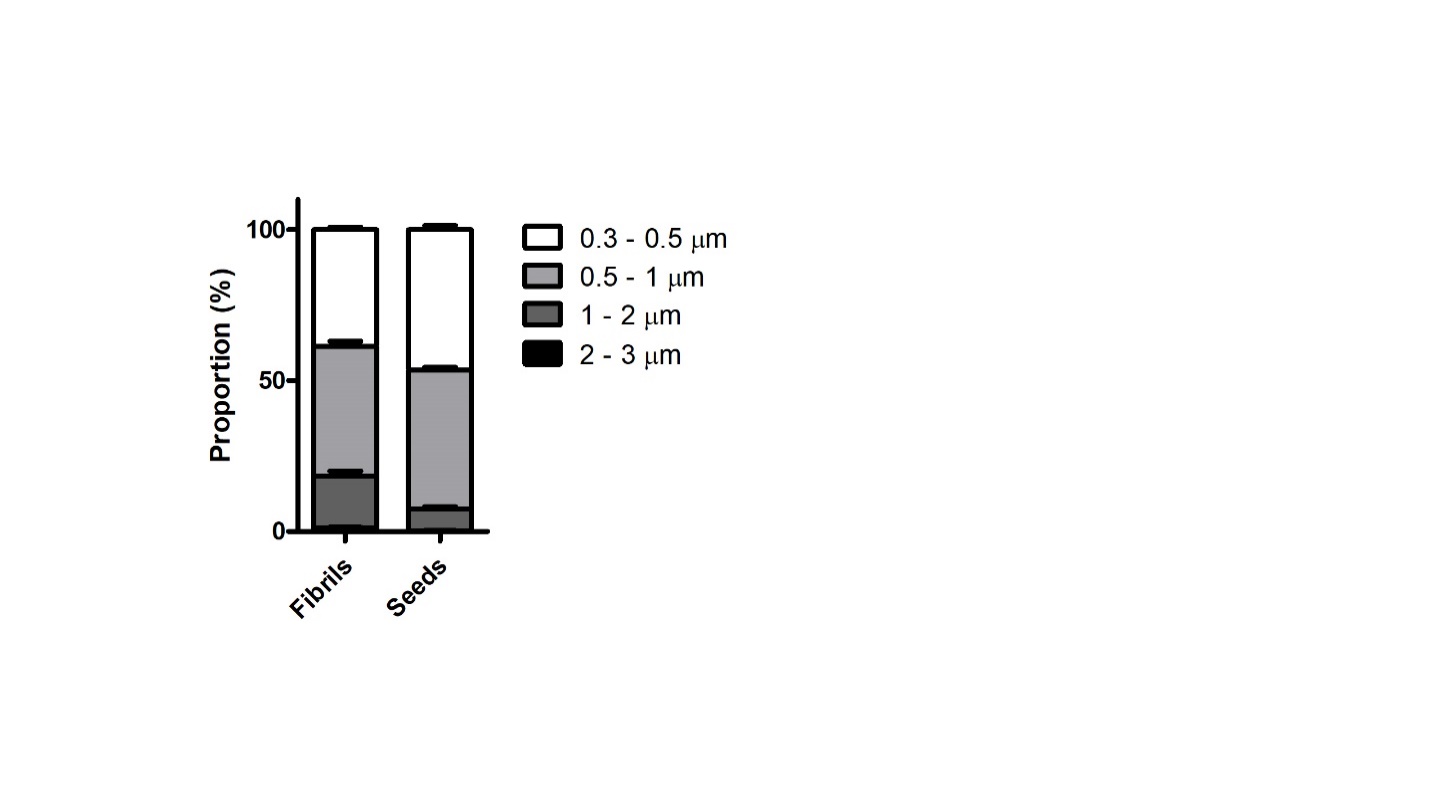


**Figure S1. The size distribution of induced aggregates in the reporter cells treated with mHTT-51Q seeds or fibrils.** The results in Fig. 1e was re-analyzed to reveal the proportion of aggregates in different sizes and plotted in the bar graph.

**FIGURE S2**


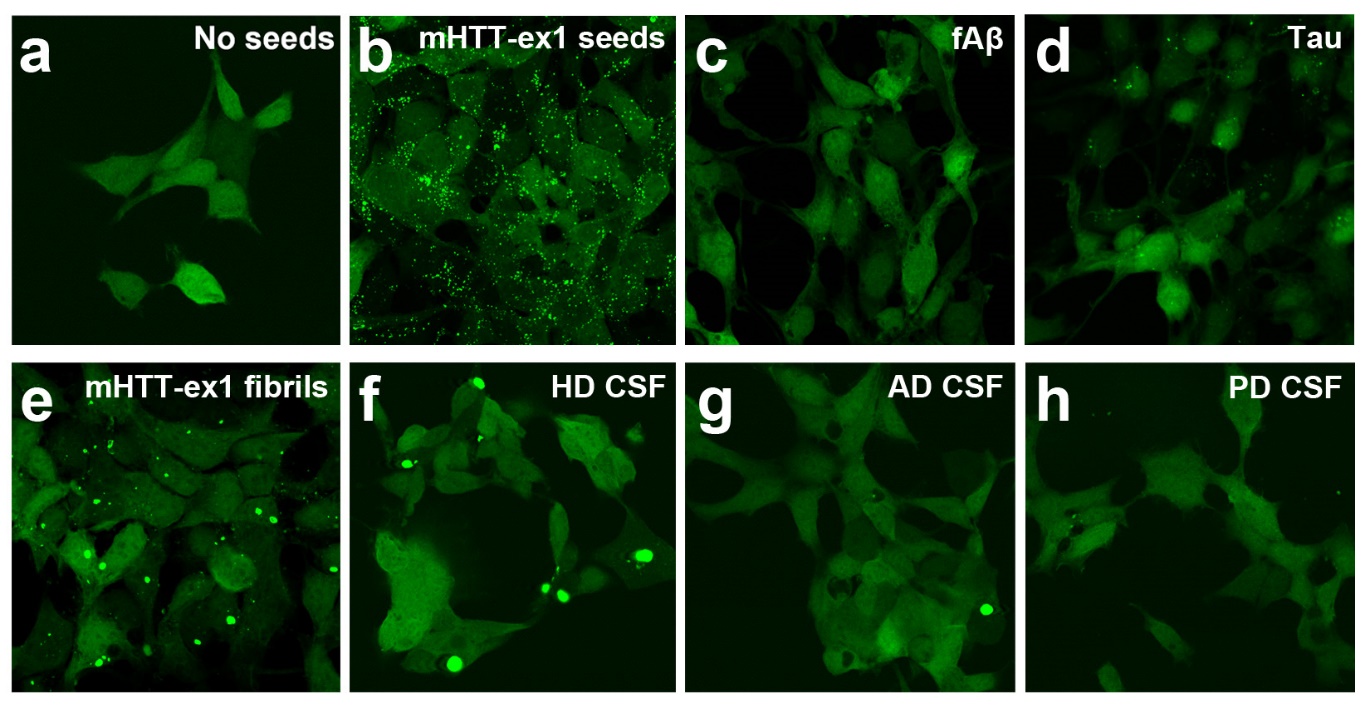


**Figure S2. The seeding assay using mHTT-ΔN17-GFP cells specifically detects mHTT seeding species in HD biosamples.** mHTT-ΔN17-GFP cells were treated with recombinant mHTT-51Q seeds (**b**) and fibrils (**e**) at 2 µg/ml, sonicated fAβ (**c**) and Tau (**d**) aggregates at 10 µg/ml, or postmortem CSF from HD (**f**), AD (**g**) and PD (**h**) subjects in a 24-well settings for 3 days. Representative confocal images demonstrated seeding induced mHTT-GFP aggregation in mHTT-ΔN17-GFP cells.

**FIGURE S3**


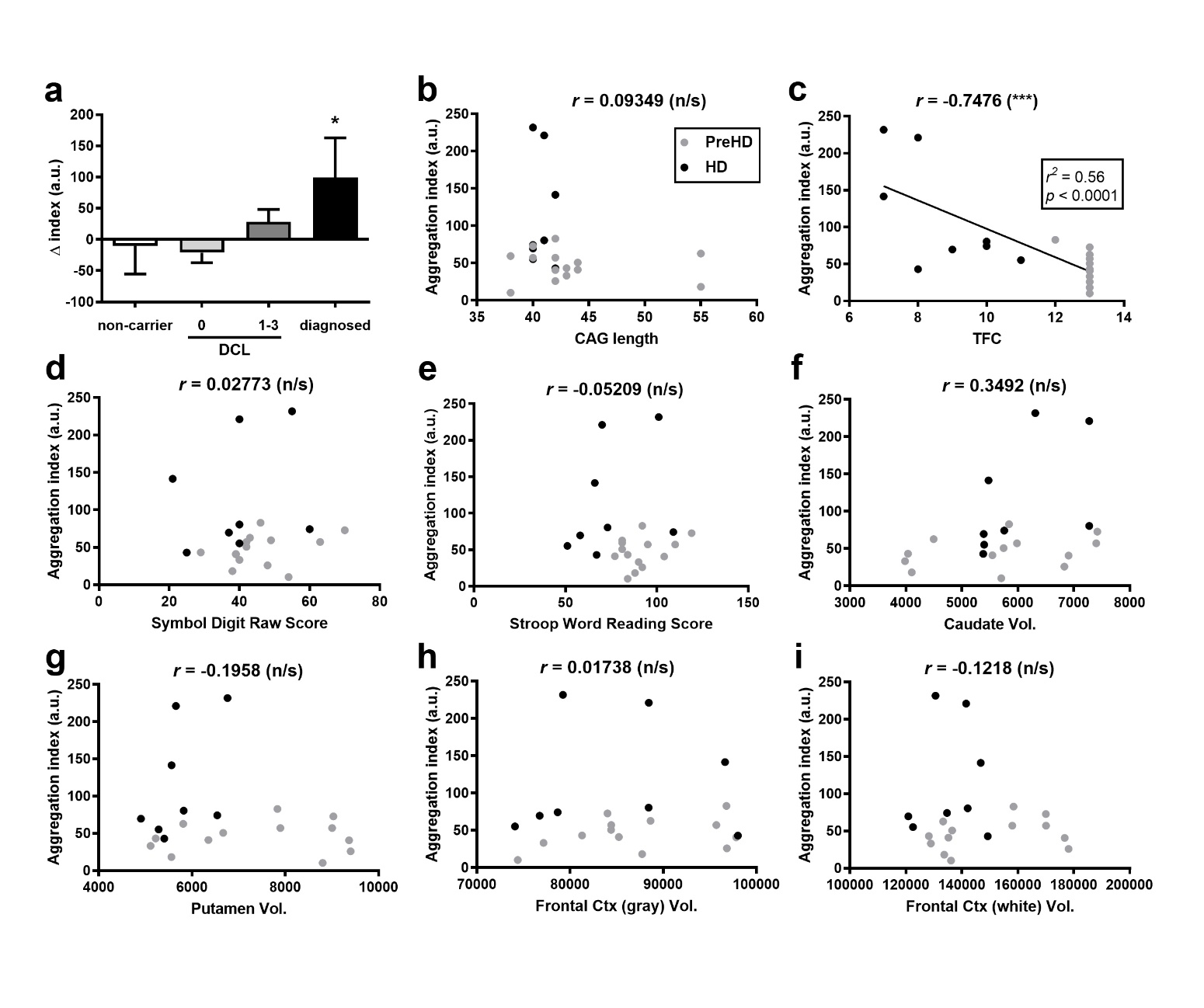


**Figure S3. Correlation between clinical measurements and mHTT seeding activities in CSF from living HD patients and mutation carriers.** Blinded CSF samples from HD patients and mutation carriers in the PREDICT-HD collection were applied to the mHTT-ΔN17-GFP cells in the 384-well format for 5 days. Confocal images were acquired with automation and batch analyzed. **a** The difference in CSF seeding activities of the two visits, one year apart, from the same individual were calculated (*n* = 3-4). The DCL score assignment in the graph is based on patient’s first visit if the DCL scores are different. **b-i** Association between CSF mHTT seeding activity and the following clinical measurements were analyzed in pairs: the CAG repeat length of the individual (**b**), total function capacity (TFC, **c**), symbol digit score (**d**), Stroop word reading score (**e**), and volumes of Caudate (**f**), Putamen (**g**), and gray (**h**) and white (**i**) matter of the frontal cortex. Individual dots are color coded by their HD diagnosis. Pearson’s correlation coefficients (*r*) were calculated and noted on the top of the plots. Statistical significance (*p* value) of the correlation is shown in parentheses. ****p*<0.001; n/s = not significant.

**Table S1. Basic characteristics of the UCLA in-house collection**

|  | HD | AD | PD |
| --- | --- | --- | --- |
| *n* | 4 | 5 | 5 |
| Males (%) | 3 (75%) | 1 (20%) | 3 (60%) |
| Age (yr) | 62.3 ± 7.8 | 81.2 ± 7.6 | 84.0 ± 7.6 |
| PMI (min) | 1,710 ± 555.3 | 1,788 ± 1,616 | 1,740 ± 1,064 |
| Pathological staging | HD3 and HD4 | Braak stage  V and VI |  |

Values are shown as mean ± s.d.

**Table S2. Basic characteristics of the NYBB cohort**

|  | Control | HD1/HD2 | HD3 | HD4 | *p* for ANOVA | *p* for HD1/HD2 vs HD3 | *p* for HD1/HD2 vs HD4 | *p* for HD3 vs HD4 |
| --- | --- | --- | --- | --- | --- | --- | --- | --- |
| *n* | 6 | 3 | 10 | 12 |  |  |  |  |
| Males (%) | 4 (66.7%) | 2 (66.7%) | 8 (80%) | 3 (25%) |  |  |  |  |
| Age (yr) | 74.5 ± 14.6 | 70.3 ± 13.6 | 58.6 ± 11.1 | 52.2 ± 10.4 | 0.0039 | 0.4413 | 0.1019 | 0.5835 |
| PMI (min) | 885.9 ± 394.1 | 1,442 ± 788.9 | 1,110 ± 507.0 | 1,470 ± 995.8 | 0.3308 | 0.9165 | 0.3180 | 0.6624 |
| CAG | - | 42.00 ± 2.65 | 44.90 ± 3.11 | 47.58 ± 5.20 | 0.1094 | 0.5658 | 0.1300 | 0.3259 |

Intergroup differences were assessed using one-way ANOVA with Turkey post-hoc analysis to determine the *p* value. Values are shown as mean ± s.d.

**Table S3. Basic characteristics of the PREDICT-HD cohort**

|  | Control | Pre-HD | HD | *p* for ANOVA | *p* for Control vs Pre-HD | *p* for Control vs HD | *p* for Pre-HD vs HD |
| --- | --- | --- | --- | --- | --- | --- | --- |
| *n* | 4 | 7 | 4 |  |  |  |  |
| Males (%) | 3 (75%) | 4 (57.1%) | 2 (50%) |  |  |  |  |
| Age | 48.1 ± 19.4 | 38.9 ± 12.1 | 62.6 ± 7.3 | 0.0019 | 0.2843 | 0.0968 | 0.0013 |
| CAG | 22.8 ± 6.9 | 43.4 ± 5.5 | 40.8 ± 0.96 | <0.0001 | <0.0001 | 0.0010 | 0.6962 |
| Aggregation index | 52.8 ± 31.04 | 46.83 ± 20.52 | 114.7 ± 74.88 | 0.0044 | 0.9491 | 0.0226 | 0.0044 |
| DCL | 0 ± 0 | 0.5 ± 1.0 | 3.8 ± 0.5 | <0.0001 | 0.2197 | <0.0001 | <0.0001 |

Intergroup differences were assessed using one-way ANOVA with Turkey post-hoc analysis to determine the *p* value. Values are shown as mean ± s.d.; Pre-HD, premanifest HD mutation carriers; HD, manifest HD patients.

**Legends for Supplemental Tables S4-S8**

**Table S4. Exhaustive analysis of covariance for the NYBB cohort.** Linear model p-values for the individual coefficients in models with aggregation index regressed on variables listed in column ‘model’. Each column shows *p*-values for one coefficient (missing whenever the variable was not included in the model), except the last 5 columns. These contain the proportion of variation of HD grade explained by the other covariates in the model, F statistic of the model, the two numbers of degrees of freedom and the corresponding p-value.

**Table S5. Exhaustive analysis of covariance with the linear mixed effect model for the PREDICT-HD cohort.** Linear mixed effect model *p*-values for aggregation index regressed on a variable of interest without covariates and with selected covariates listed in column Covariates. DCL.combined refers to DCL with levels 1 and 2 combined into a single level (labeled 1.2) and non-carriers and DCL level 0 carriers combined into level 0. Column ‘DCL.combined’: p-values of the coefficient for DCL level 4 in a model with variable of interest being DCL treated as a factor; column ‘DCL.combined.1.2.vs.0’: p-values of binary indicator of DCL 1 or 2 vs. 0 (and missing otherwise) being the variable of interest; column ‘DCL.combined.3.vs.0’: p-values of binary indicator of DCL 3 vs. 0 (and missing otherwise); column ‘DCL.combined.4.vs.0’: p-values of binary indicator of DCL 4 vs. 0 (and missing otherwise); column ‘cap’: p-values for the CAP score being the variable of interest; column ‘cag’: p-values for the CAG length of the expanded allele being the variable of interest; column ‘height_valid’: *p*-values for physical height being the variable of interest; column ‘DCL.PVE’: the proportion of variation of DCL (treated as a numeric variable) explained by the covariates.

**Table S6. Regression analysis using the linear mixed effect model on DCL scores vs. other variables for the PREDICT-HD cohort.** Linear mixed effect model *p*-values for the individual coefficients in models with aggregation index regressed on variables listed in column ‘model’. Each column shows *p*-values for one coefficient (missing whenever the variable was not included in the model), except the last column which contains the proportion of variation of DCL explained by the other covariates in the model.

**Table S7. Shapiro-Wilks normality test for residuals of a mixed effect linear model of the PREDICT-HD cohort.** Results of Shapiro-Wilks normality test for residuals of a mixed effect linear model. Each column contains the Shapiro-Wilks test *p*-values for a model regressing aggregation index on the variable in the column name plus covariates listed in the column Covariates.

**Supplemental Table S8. Regression analyses of the aggregation index and clinical scores for the PREDICT-HD cohort.** Results of regressing aggregation indices on clinical variables without covariates. Column ‘*p* value for aggregation score’; linear mixed effect model p-values for each variable; column ‘Bonferroni’: *p*-values corrected for multiple testing using Bonferroni correction; column ‘Shapiro Wilks normality test p-value’: *p*-values returned by the Shapiro-Wilks normality test of the residuals of the model.
